# Effect of Imposing Spatial Constraints on Low Molecular Weight Gels

**DOI:** 10.1021/acs.biomac.3c00559

**Published:** 2023-08-18

**Authors:** Max J. S. Hill, Ana M. Fuentes-Caparrós, Dave J. Adams

**Affiliations:** School of Chemistry, University of Glasgow, Glasgow G12 8QQ, U.K.

## Abstract

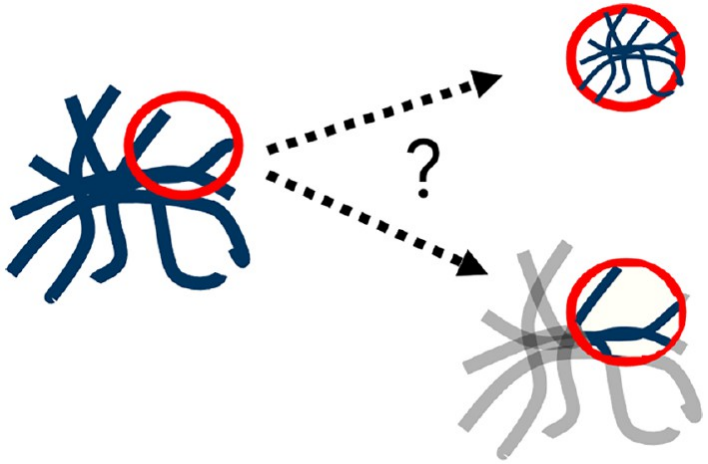

We outline the effect of imposing spatial constraints during gelation on hydrogels formed by dipeptide-based low molecular weight gelators. The gels were formed via either a solvent switch or a change in pH and formed in different sized vessels to produce gels of different thickness while maintaining the same volume. The different methods of gelation led to gels with different underlying microstructure. Confocal microscopy was used to visualize the resulting microstructures, while the corresponding mechanical properties were probed via cavitation rheology. We show that solvent-switch-triggered gels are sensitive to imposed spatial constraints, in both altered microstructure and mechanical properties, while their pH-triggered equivalents are not. These results are significant because it is often necessary to form gels of different thicknesses for different analytical techniques. Also, gels of different thicknesses are utilized between various applications of these materials. Our data show that it is important to consider the spatial constraints imposed in these situations.

## Introduction

Gels are inherently dynamic materials. This is especially true for supramolecular gels.^1^ Here, the reversible nature of the noncovalent interactions that underpin the molecular architecture of the gelators, the molecules that self-assemble to form the network that gives rise to this class of materials, makes them responsive to external stimuli and conditions.^2^ The gelation process itself requires specific conditions to balance the solubility of gelator molecules and drive them to self-assemble, giving a class of material that is highly sensitive to the conditions under which it is formed.^3−8^

**Figure 1:**
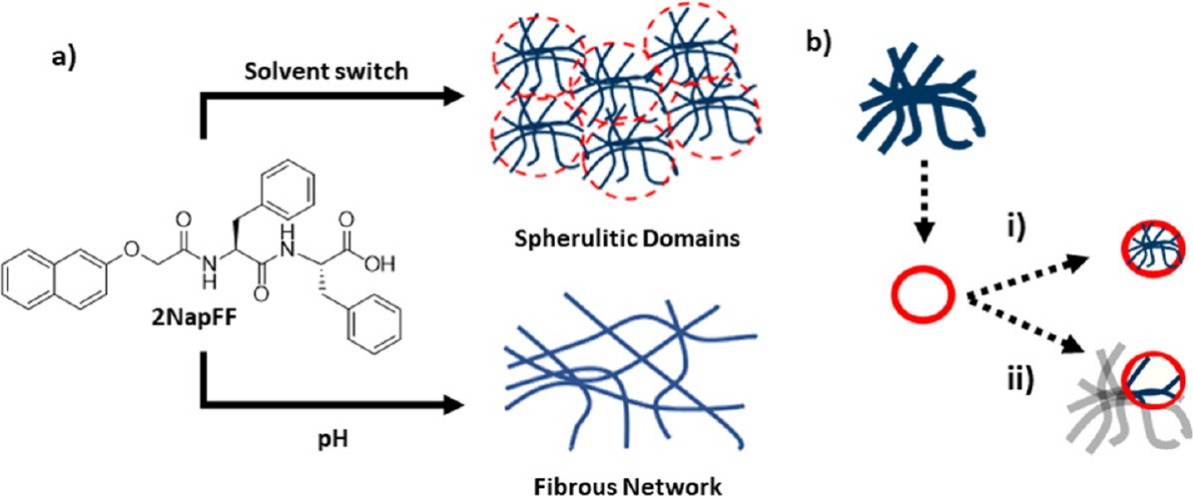
(a) Gelation pathways for 2NapFF. Slow acidification via GdL forms a uniform fibrous network (bottom), while a DMSO:H_2_O solvent switch can form spherulitic domains (top). (b) Cartoon diagram demonstrating potential effects of vessel size constraint (red circle) on an ideal spherulite (blue). Either (i) a smaller spherulite could form within a confined space or (ii) an incomplete part of a spherulite could form within a confined space.

It is therefore unsurprising that different gels can be formed from different gelators. Changing the building blocks from which the gels are formed results in a different material. Potentially less intuitive is the ability to form materials from the same gelator through altering the preparation process, leading to gels presenting distinct mechanical, or other, properties.^5,9−14^ It has been demonstrated that different gelation triggers as well as modifications to or variations within the same trigger can all lead to distinct materials being produced from the same starting gelators.^6,7,9−11,15−17^ Often this is due to subtle changes to the underlying solid-like gelator network and the microstructure it presents.^18−21^ For example, Huang et al. and Almohammed et al. demonstrated that it was possible to achieve distinct microstructures comprising completely different morphologies in materials produced from the same gelators by changes in temperature.^15,22,23^ This was built upon by Chen et al. and Dudukovic et al., who both demonstrated that microstructure morphology could be altered by varying solvent ratios within a solvent-switch gelation trigger (Figure 1a).^6,16^ These studies support the aforementioned key idea that gelation within this class of materials is affected by a vast range of variables.

While it has been demonstrated that vessel size can influence protein aggregation and thus amyloid fibril formation,^24^ to the best of our knowledge the effect that the vessel in which low molecular weight gels are formed may have has yet been unexplored. Gels typically adopt the shape of the container in which they are formed, which necessitates samples being formed in a range of vessels to be suitable for the various methods of characterization, such as NMR tubes or capillaries for scattering.^25−27^ This results in gels being formed in different shapes and on different scales. For largely uniform materials, this would not be expected to have a significant impact. But the above studies have shown that some low molecular weight gelator (LMWG)-based gels do contain an underlying network comprising more compartmentalized building blocks, tethered to one another by interconnecting links, leading to a nonuniform morphology.^6^ For these systems, it seems more likely that the shape and scale in which the gel is formed to have an effect. The constituent building blocks within these networks, spherulite-like domains, are of a set size under a specific set of gelation conditions and parameters.^16^

Typically, gels are formed on scales many orders of magnitude greater than those of the underlying domains. However, with this class of materials being idealized for more modern biomedical applications, such as providing structural supports for tissue engineering or cell culture, samples could feasibly be produced on scales approaching, or even encroaching, that of these domains, meaning their size becomes significant.^28,29^ A question is raised about how these materials behave under such spatial constraints. Smaller domains may be formed much the same way a plant within a smaller plot will only grow to the space available (Figure 1b(i)). Alternatively, partially formed domains may instead be created, with bundles of fibers growing to the same scales, but instead forming “incomplete” spherulites (Figure 1b(ii)).

A deeper understanding of how these networks behave when confined is vitally important for the use of gels within model protocells, where they can be used to mimic cell membranes or intracellular matrices.^30,31^ The design and limitations of these systems would be heavily influenced by the morphology of the gelator network underpinning these gels.

## Materials and Methods

**Figure 2:**
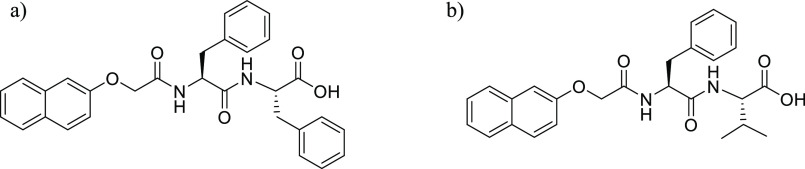
Chemical structures of 2-naphthylmethyl ether (Nap) protected (a) diphenylalanine (2NapFF) and (b) phenylalanine–valine (2NapFV) dipeptide low molecular weight gelators.

2NapFF (F = phenylalanine) and 2NapFV (V = valine) were synthesized as previously outlined (chemical structures shown in Figure 2).^11,32−34^ DMSO was purchased from Fisher Scientific and Nile Blue A was purchased from Sigma-Aldrich, with all used as received. Deionized water was used throughout.

**Figure 3:**
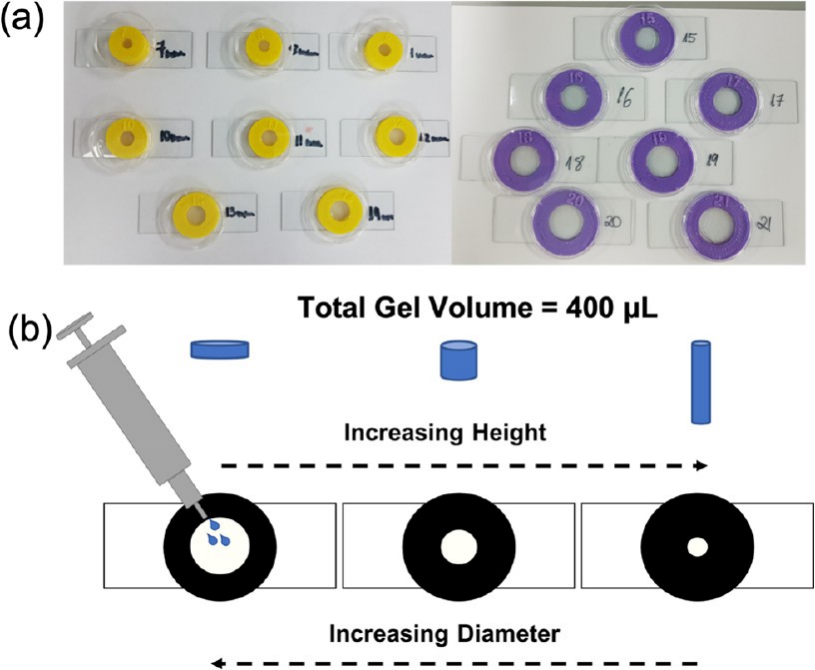
(a) Photograph of the 3D printed ring-shaped vessels used to prepare gels of differing thickness. Ring diameter (mm) is labeled on each ring/slide and increases in 1 mm increments from 7 to 21 mm. Clear plastic lids from Greiner CellStar cell culture dishes were used as shown to reduce potential evaporation during gelation. (b) Cartoon of different thickness gels being formed within different diameter ring-shaped vessels.

The same volume of gel (400 μL) was formed within ring-shaped vessels of different diameters, resulting in gels of different thickness. These vessels were created by 3D printing plastic rings of different diameters (from 7 to 21 mm) and adhering these to standard borosilicate glass microscope slides with Araldite two-part glue (Figure 3). This allowed for gels of varying thickness to be formed (ranging from 1 to 10 mm), as shown in Table 1.

**Table 1:** Vessel Diameters and Corresponding Calculated Gel Thickness

cavity ring diameter (mm)	calculated gel thickness (mm)	gel volume (μL)
7	10.39	400
8	7.96	400
9	6.29	400
10	5.09	400
11	4.21	400
12	3.54	400
13	3.01	400
14	2.60	400
15	2.26	400
16	1.99	400
17	1.76	400
18	1.57	400
19	1.41	400
20	1.27	400
21	1.15	400

### Forming Gels of Differing Thickness

For the solvent-triggered gels, a gelator was dissolved in DMSO (25 mg/mL). 80 μL of this stock solution was pipetted into the desired ring-shaped vessel, with care taken to evenly cover the bottom of the vessel. 320 μL of water was then added via a 1 mL automatic pipet as a single aliquot to the center of the vessel, forming a homogeneous gel (400 μL, 5 mg/mL) at a DMSO volume fraction (φ_DMSO_) of 0.2.

For the pH-triggered gels, a basic aqueous gelator solution was formed by the addition of gelator to water (5 mg/mL) and 1 mol equiv of 0.1 M NaOH, before being left to stir overnight. This aqueous stock solution was then adjusted to pH 10.5 by using aliquots of 0.1 M NaOH. A 400 μL portion of this aqueous stock solution was pipetted into a vial containing GdL (8 mg/mL), mixed thoroughly with a spatula for 5 s, and quickly transferred to the center of the desired ring-shaped vessel to form a homogeneous gel (400 μL, 5 mg/mL). Samples were left to gel overnight within sealed Petri dishes containing wet tissue to prevent samples drying.

### Nonuniform Vessels

**Figure 4:**
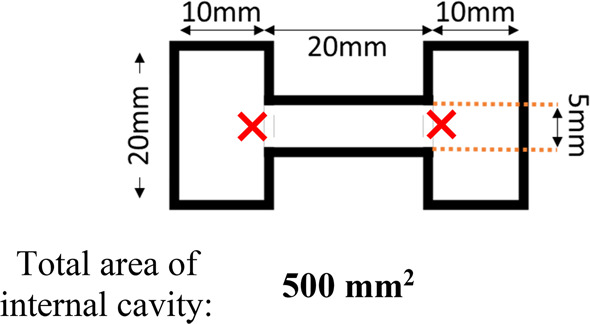
Schematic diagram of custom 3D printed gel molds in a dumbbell shape. Dimensions are given in millimeters and represent those of the internal cavity in which to form gel, not to scale. The positions from which water aliquots were pipetted in are highlighted with red crosses.

To subject portions of a single gel to different spatial constraints, 2NapFV and 2NapFF gels were formed within 3D printed dumbbell-shaped vessels (Figure 4).

As for the ring-shaped vessels, an aliquot of gelator stock solution in DMSO (25 mg/mL) was pipetted into the mold and spread evenly to give uniform coverage. This was then followed by an aliquot of water administered as two equal aliquots at different positions within the mold. This gave gels with a final gelator concentration of 5 mg/mL and φ_DMSO_ of 0.2. Gels were formed at volumes resulting in a final gel height of 2 mm in each mold.

For the pH-triggered gels, a basic aqueous gelator solution was formed by the addition of gelator to water (5 mg/mL) and 1 mol equiv of 0.1 M NaOH, before being left to stir overnight. This stock solution was then adjusted to pH 10.5 using 0.1 M NaOH. An aliquot of this stock solution was transferred to a vial containing GdL (8 mg/mL), mixed thoroughly with a spatula for 5 s, and pipetted into the dumbell-shaped mold. Samples were then left to gel overnight within sealed Petri dishes containing wet tissue to prevent samples drying. Gels were formed at volumes resulting in a final gel height of 2 mm in each mold.

### Confocal Microscopy

A Zeiss LSM 710 confocal microscope fitted with Zeiss N-Achroplan 10× and LD EC Epiplan NEUFLUAR 50× (0.55 DIC) objectives was used for confocal fluorescence microscopy imaging. Nile Blue A dye (0.1 wt % aqueous solution, 2 μL per mL of gel) was incorporated into gels to allow for imaging. The dye was added either dissolved within the water aliquot for the solvent-triggered gels or directly into the aqueous stock solution before GdL addition for the pH-triggered gels. Gels were prepared in custom ring-shaped vessels with glass microscope slide bottoms. Nile Blue fluorescence was achieved by excitation with a 634 nm He–Ne laser and emission detected between 650 and 710 nm. Multiple images were captured for each sample to ensure reproducibility.

### ImageJ Analysis

ImageJ image processing software was used to quantify the perimeters of assigned spherulitic domains within gels. Generally, 5 different spherulites were chosen and measured across at least 2 images of each sample, with the perimeters analyzed with ImageJ. Exemplified perimeter assignments for solvent-triggered 2NapFV are shown in Figure S1 of the Supporting Information. For microstructures presenting less distinct spherulites, domain boundaries were determined by eye as the point away from the densely populated centers where fiber density noticeably decreased to sparser spanning fibers.

### Cavitation Rheology

A bespoke instrument produced in-house was used for cavitation rheology measurements as described previously (Figure S3).^35,36^ Air was pumped at a rate of 0.5 mL/min from a 10 mL Hamilton 1000 gastight syringe. For measurements, the tip of the needle (22 gauge) was set to a depth of 1 mm below the detected surface of the sample. The tip of the needle was positioned centrally in the sample. The critical pressure (*P*_c_) was defined as the maximum pressure achieved within the system before the cavitation bubble formed with the sample burst, and the pressure subsequently fell. Measurements were performed on three separate samples prepared in triplicate.

Use of this technique is limited by a minimum gel thickness, at which reliable measurements can be achieved. A 1 mm working depth was used during the experiment, so very thin gels approaching this depth would not provide reproducible data due to edge effects. Here, growth of the cavitation bubble is disturbed or impeded by the close proximity to the bottom of the container, making the observed *P*_c_ values unreliable. We found that the minimum gel thickness at which cavitation rheology could be reliably performed was 2.5 mm (corresponding to a 14 mm diameter vessel). Therefore, only gels thicker than 2.5 mm (vessel diameter ≤14 mm) could be analyzed via cavitation rheology using our existing setup.^36^ Edge effects were also expected to affect measurements taken close to the edge of the sample. Here, the solid walls of the vessel could interfere with or impede the growth of the cavitation bubble.

## Results and Discussion

This work demonstrates the effect of imposing spatial constraints during gelation on two low molecular weight gels. This was initially achieved by forming separate gels of different thickness while maintaining the same volume, followed by single gel samples in vessels with compartments of different size. Gels of 2NapFF and 2NapFV (structures shown in Figure 2), both from a known family of low molecular weight gelators (LMWGs),^32,37,38^ were selected to highlight this. We chose these specific gelators on the basis of their clear differences in microstructure, as evidenced by confocal microscopy (see below). Different microstructures are further formed within this class of materials through the use of different methods of triggering gelation, such as pH or solvent switching.^11,32,39,40^ The latter results in spherulite-like domains, reminiscent of those seen in organogels, when appropriate solvent ratios are implemented.^15,41^

Prospective applications for these materials within the cell culture and tissue engineering fields mean it is important to explore and understand their response to spatial constraints that may be imposed within these applications.^37,38,42,43^ To this end, gels of different thickness were formed and then characterized by confocal microscopy and cavitation rheology. To achieve this, rings from 7 to 21 mm in diameter were 3D printed and bonded to microscope slides. By forming the same volume of gel within each ring-shaped vessel, gels of different thickness (ranging from 1.15 to 10.39 mm) were produced (Figure 3b). To ensure that these results were representative, multiple images and measurements were taken for each sample at different points throughout the gel. To ensure that observed differences were a direct result of differences in vessel size, gels of different volume, and therefore height, were formed in the same sized vessels (Figure S2). No significant differences in microstructure were observed between the gels of different height. This also demonstrates that the varied surface contact with surfaces of different hydrophilicities, i.e., glass and plastic, within the different vessels is not a contributing factor to observed differences in microstructure.

### 2NapFF

**Figure 5:**
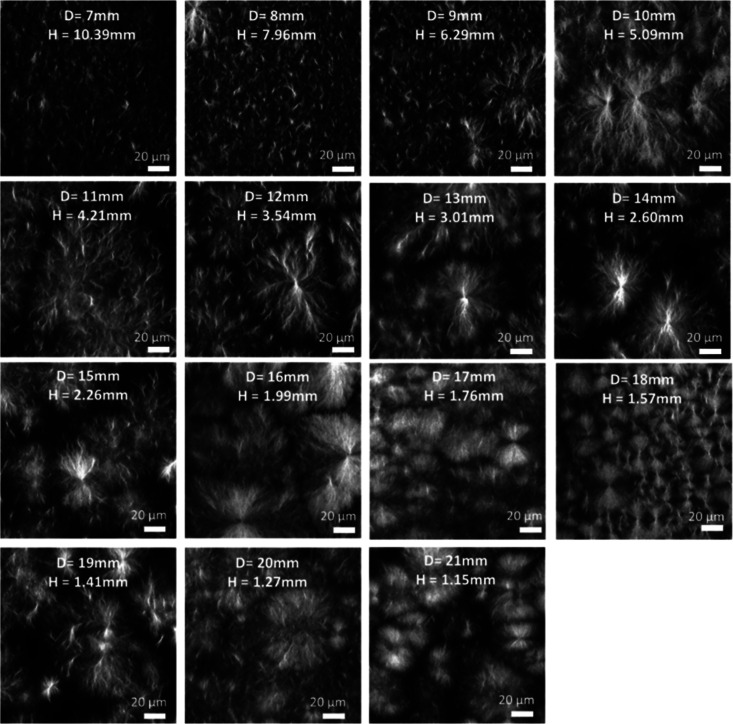
Confocal images of solvent-triggered 2NapFF gels prepared in 3D-printed ring-shaped vessels with different diameters. All gels were prepared at 5 mg mL^–1^ using a φ_DMSO_ = 0.2 and a total gel volume of 400 μL. Nile Blue A dye was incorporated pregelation (0.1 wt % aqueous solution at 2 μL per mL of gel). Scale bars (white) represent 20 μm in all cases. *H* = height and *D* = vessel diameter.

Initially we focus on gels formed by a solvent switch, which lead to gels with an underlying microstructure consisting of spherulitic domains of fibers.^6^ It is expected that thicker gels will allow larger constituent spherulitic domains to grow as more room is available for expansion and growth during the nucleation and growth phase separation process that creates this microstructure.^15,41^ Here we show the changes of these domains within 2NapFF gels as the thickness of the gel is varied. Figure 5 shows the microstructure of solvent-triggered 2NapFF gels prepared at different thicknesses, with differences in the spherulite sizes observed. To quantify these domains, we used ImageJ to measure the perimeters of the observed spherulitic structures (Table S1). It is evident from these images that moving between different thicknesses of gels affects the spherulite-like domains within. For the vessel sizes tested, the spherulite size initially increases as the gels become thicker, up to 5 mm, after which spherulites decrease in size before hitting a plateau at a perimeter roughly 200 μm in size (Figure 5). We highlight that we focus here on using confocal microscopy to understand how the microstructure and network is changing. Techniques such as TEM focus on the self-assembled structures as opposed to the microstructure. Additionally, TEM often images artifactual changes in the self-assembled structures due to drying issues,^44^ and cryo-TEM is often unable to image the network due to the requirement for trapping the sample to be imaged in a thin film (typically <300 nm).^45^

**Figure 6:**
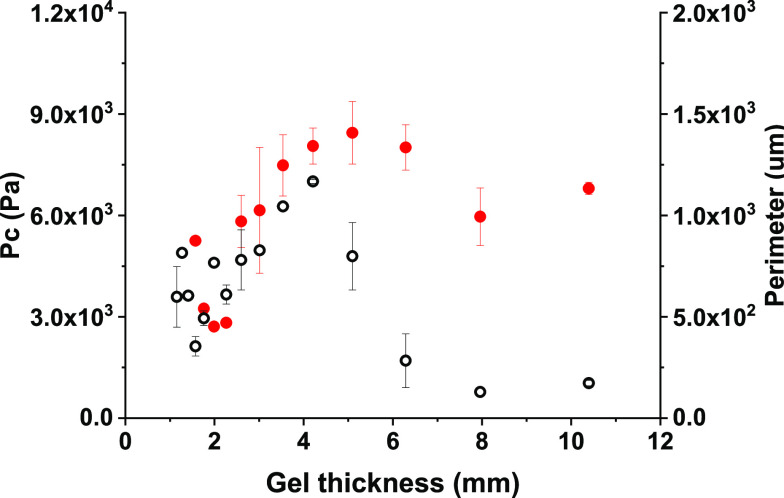
Correlation between the critical pressure (*P*_c_) and spherulite perimeters within 2NapFF gels of different thickness. The perimeter is represented with black hollow circles, and the critical pressure is represented with red circles. The error bars represent the standard deviations of three repeated measurements.

The mechanical properties of these gels were characterized by needle-induced cavitation rheology (Figure 6).^46^ This is a form of microrheology utilizing the cavitation effect to probe localized mechanical properties of a material.^35,36,46^ Briefly, air is pumped through a needle inserted into the sample, forming a cavitation bubble at the tip. Through its growth, this bubble exerts outward pressure, thus applying stress to the surrounding material. This bubble bursts as the material encapsulating it fails, at a point deemed the critical pressure (*P*_c_). These values can be compared across a sample, between materials, and even correlated to viscoelastic moduli measured via oscillatory rheology.^35,36^ While the latter is typically a standard for characterization of this class of materials, it is impossible to carry out consistently in this context as to allow for accurate comparisons of mechanical properties between materials; a consistent sample must be tested.

Figure 6 shows the correlation between the critical pressure and spherulite perimeters within gels of different thickness. Starting from the thinner gels, initially both *P*_c_ and spherulite perimeter show a general increase up to a gel thickness of around 4 mm, after which both then drop as the gel thickness further increases. This appears to then be followed by a plateau, as *P*_c_ and the spherulite perimeter remain of similar values for the thicker gels. The lowest critical pressure observed for the thinnest gels may simply be due to less gel supporting the cavitation bubble being grown, tending toward an edge effect in which the nonelastic wall of the vessel interferes with continued growth of the cavitation bubble.

### 2NapFV

**Figure 7:**
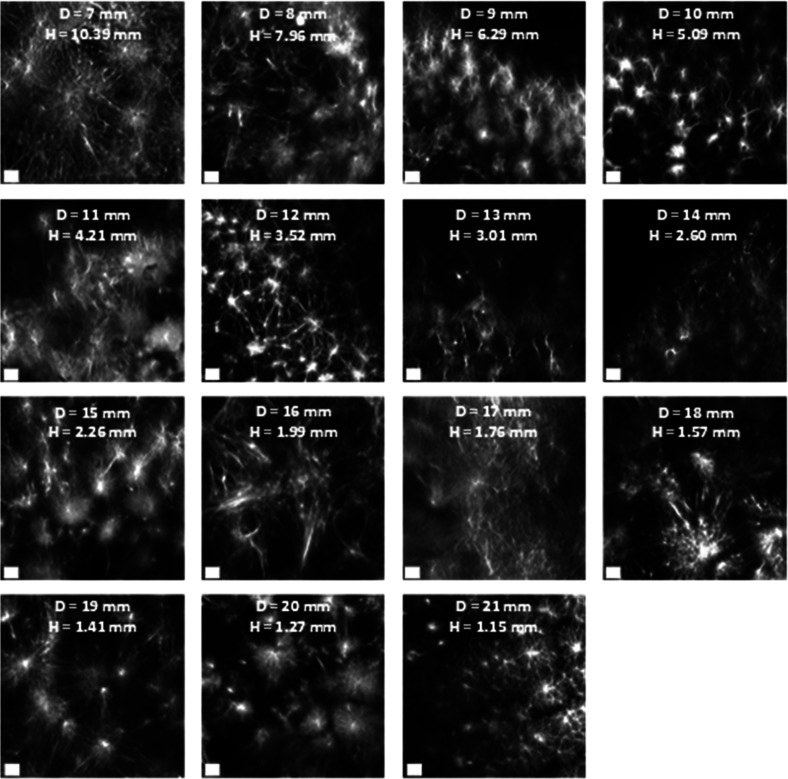
Confocal images of the solvent-triggered 2NapFV gels prepared in 3D-printed ring-shaped vessels with different diameters. All gels were prepared at 5 mg mL^–1^ using a φ_DMSO_ = 0.2 and a total gel volume of 400 μL. Nile Blue A dye was incorporated pregelation (0.1 wt % aqueous solution at 2 μL per mL of gel). Scale bars (white) represent 50 μm. *H* = height and *D* = vessel diameter.

This experiment was repeated with 2NapFV gels to see if the trends shown for 2NapFF applied to a related gelator. 2NapFV also produces gels with a spherulitic microstructure when produced via a solvent-switch gelation trigger below a critical solvent ratio. As before, gels of different thickness were formed in ring-shaped vessels of varying diameter and characterized via confocal microscopy and cavitation rheology. Figure 7 shows the microstructure presented by the solvent-triggered 2NapFV gels of different thickness. Initially spherulite size decreases as the gel thickness increases up until 6 mm, at which point spherulite size begins to increase again. This contrasts with the trend seen for the 2NapFF gels.

**Figure 8:**
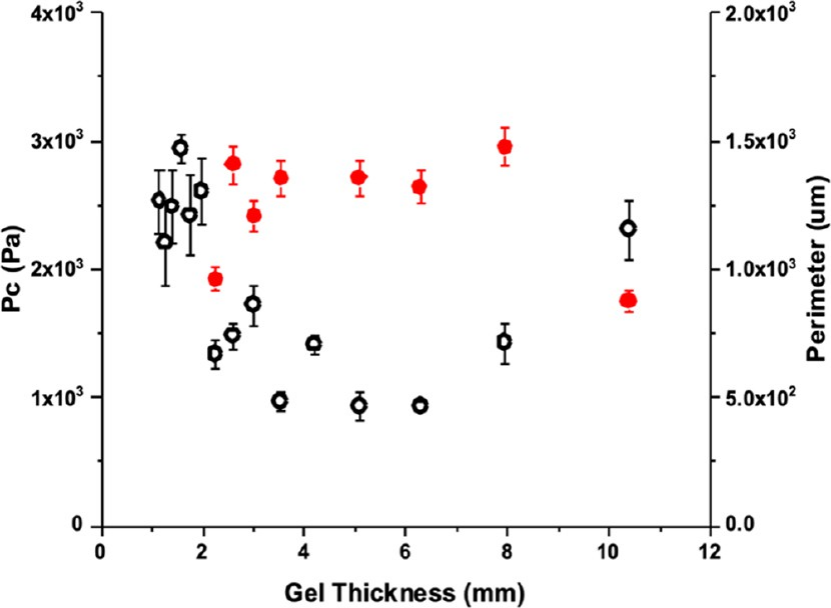
Correlation between the critical pressure (*P*_c_) and spherulite perimeters within solvent-triggered 2NapFV gels of different thickness. The perimeter is represented with black hollow circles and the critical pressure with red circles. The error bars represent the standard deviation of three repeated measurements.

Figure 8 overlays the spherulite perimeters with the critical pressures observed through cavitation rheology for solvent-triggered 2NapFV gels. An apparent inverse relationship between the spherulite size and *P*_c_ values is seen for this system, with the largest spherulites presenting the lowest *P*_c_ and the highest *P*_c_ values seen for the smallest spherulites. The lowest critical pressure observed for the thinnest gels may simply be due to less gel supporting the cavitation bubble being grown, tending toward an edge effect in which the nonelastic wall of the vessel interferes with continued growth of the cavitation bubble. The spherulites seen within the thickest 2NapFV gels examined were significantly larger than those seen in 2NapFF gels of the same size.

The spherulite-like domains formed in these solvent-triggered gels are produced via a nucleation and growth process wherein the underlying network grows from dispersed droplets of gelator aggregated in organic solvent.^3,6,15,47−49^ Some of these nucleation points then develop outward at the expense of others through Ostwald ripening, leading to a microstructure that is not uniform and contains denser clusters of gelator fibers surrounded by more sparsely populated regions containing spanning fibers linking adjoining clusters.^4^ Due to the radial propagation during gelation and heterogeneous composition of these networks, it is unsurprising that they are affected by formation within different environments.

### pH-Triggered Gels

For both 2NapFV and 2NapFF, gels can also be formed via a pH trigger. Here, a solution is initially prepared at high pH, and then gelation occurs when the pH is decreased. To carry out the decrease in pH, we used the hydrolysis of glucono-δ-lactone (GdL),^50^ which can be used to form pH-triggered gels that present a near uniform fibrous microstructure,^11^ with gelation occurring over a longer time scale for these systems compared to the solvent-triggered counterparts.^7^ 2NapFF and 2NapFV GdL-triggered gels were also formed within ring-shaped vessels of varying diameter to compare the response of these pH-triggered systems to their solvent-triggered counterparts.

**Figure 9:**
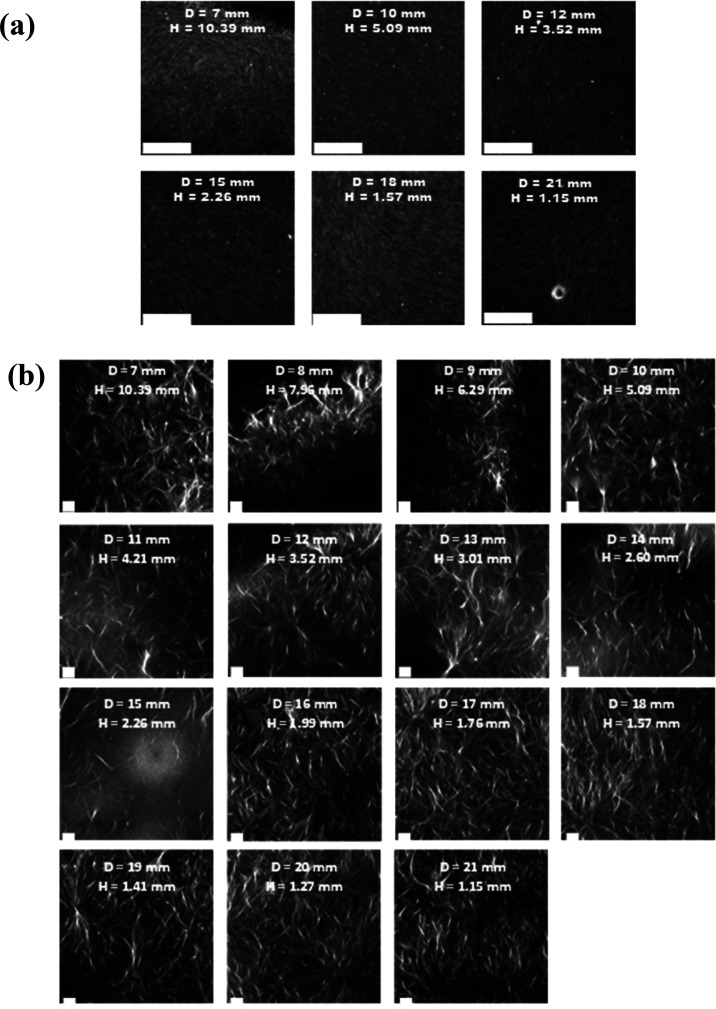
Confocal microscopy images of GdL-triggered (a) 2NapFF and (b) 2NapFV gels prepared in 3D-printed ring-shaped vessels with different diameters. All gels were prepared at 5 mg mL^–1^ using 8 mg mL^–1^ GdL and a total gel volume of 400 μL. Nile Blue A dye was incorporated pregelation (0.1 wt % aqueous solution at 2 μL per mL of gel). Scale bars (white) represent 50 μm. *H* = height and *D* = vessel diameter.

Figure 9 shows the microstructures presented by GdL-triggered gels formed within differently sized vessels. A consistent fibrous network is present within both gels, with little variation observed between gels of different thickness based on the same gelator. Both fiber size and density appear to remain relatively self-consistent for each material regardless of vessel size. The fibers comprising the 2NapFV gelator network are thicker and less densely packed than those forming the 2NapFF network.

These underpinning fibrous networks grow progressively denser over time after the pH has fallen below the p*K*_a_ of the gelator until gelation is complete.^51^ This process is not diffusion limited due to the rate of pH decrease being slow compared to the rate of GdL diffusion, which allows these gels to form consistently regardless of the space available during gelation, unlike the solvent-triggered counterparts.

**Figure 10:**
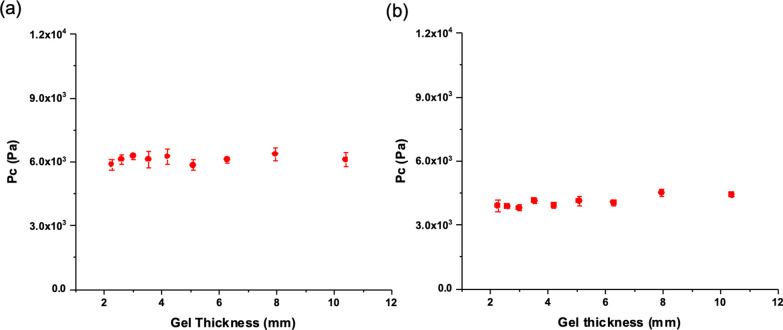
Measured critical pressures (*P*_c_) of GdL-triggered (a) 2NapFF and (b) 2NapFV gels of different thickness. The error bars represent the standard deviation of three repeated measurements.

Cavitation rheology was used to determine critical pressure values for both 2NapFF and 2NapFV GdL-triggered gels of differing thicknesses (Figure 10). As expected, given the similar microstructures observed within each set of gels (Figure 9), these were consistent for each gel, with no significant differences observed in the magnitude of *P*_c_. The 2NapFF GdL gels presented a higher average *P*_c_ value than those of 2NapFV. These pressures indicate that the scale of formation does not impact localized mechanical properties within these pH-triggered systems.

### Multisize Vessels

**Figure 11:**
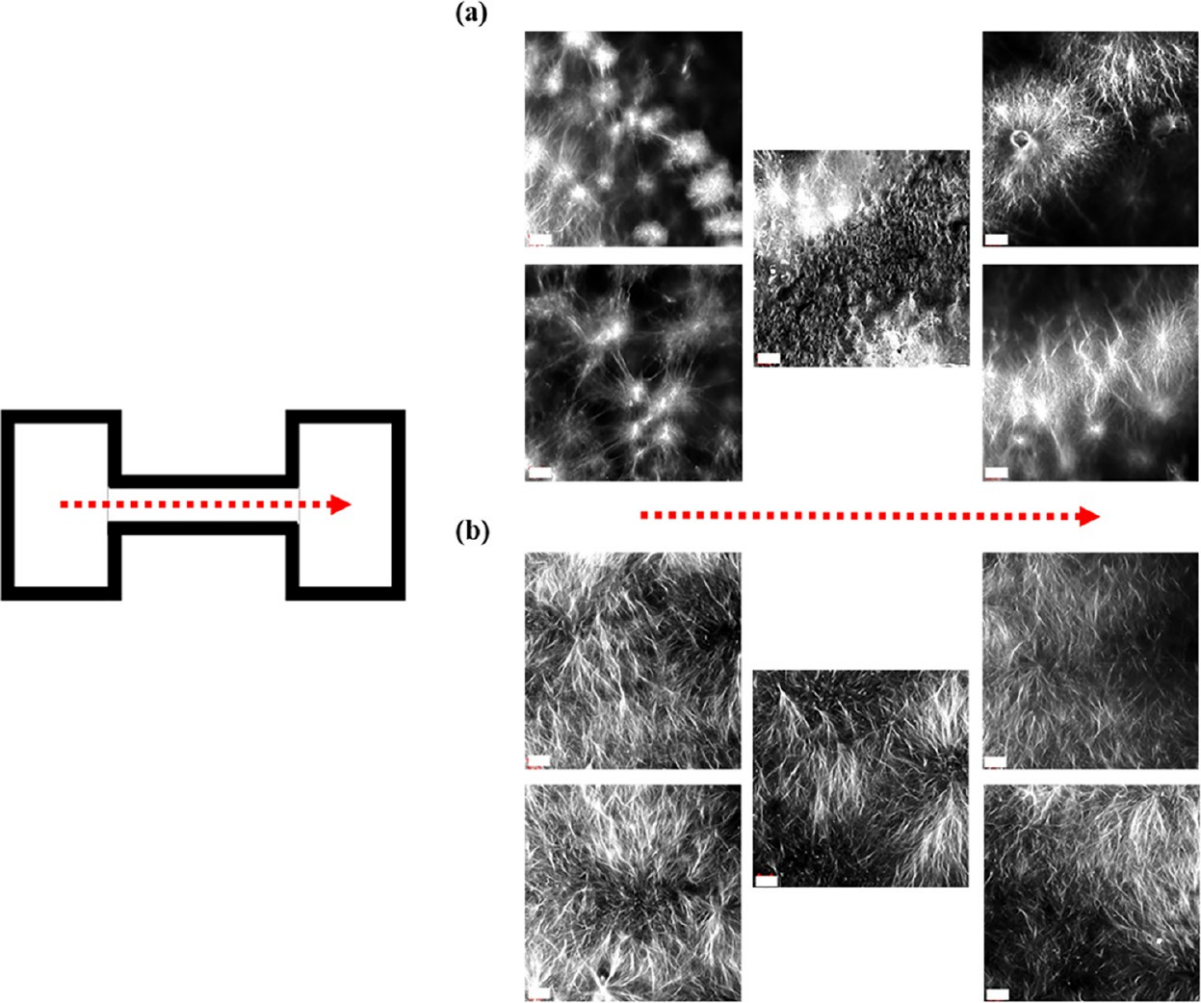
Confocal images of (a) solvent- and (b) pH-triggered 2NapFV gels prepared in 3D-printed dumbbell-shaped vessels (Figure 4). All solvent-triggered gels were prepared at 5 mg mL^–1^ using φ_DMSO_ = 0.2. All pH-triggered gels were prepared at 5 mg mL^–1^ using a GdL concentration of 8 mg mL^–1^. Gel volumes were chosen to produce 2 mm tall gels. Nile Blue A dye was incorporated pregelation (0.1 wt % aqueous solution at 2 μL per mL of gel). Scale bars (white) represent 50 μm. The direction of imaging is indicated with red arrows.

To clearly demonstrate the effect of applying size constraints on gels displaying a spherulite-like microstructure, gels were formed in vessels containing portions of different widths. This was achieved via a dumbbell-shaped mold (Figures 4 and 11). By forming a single solvent or pH-triggered gel within these molds, material in different parts of the vessel would be subject to different spatial constraints. The confocal images of the 2NapFV solvent-triggered gel shows significantly differing microstructures within the gel formed within the different parts of the mold (Figure 11a). Significantly larger, more developed spherulite-like domains are seen within the gels contained within the wider ends of the mold. In the thinner central portion, the gel consists of smaller, more distinct spherulites similar to those typical of 2NapFF gels (Figure 5). This was compared against pH-triggered equivalents.

A GdL-triggered 2NapFV gel was then formed within the same vessel and imaged in an identical manner (Figure 11b). In line with the above observations for separate pH-triggered gels formed at different diameters, a consistent microstructure was presented throughout this gel. A uniform fibrous gelator network was present in both the wider ends and thinner central portion of the gel, with no clear differences seen between these regions (Figure 11b). This confirms the sensitivity of the solvent-triggered low molecular weight gels of this type to imposed spatial constraints while also showing the pH-triggered counterparts show unaffected microstructure.

## Conclusions

The differences observed in the morphology and mechanical properties of the solvent-triggered 2NapFF and 2NapFV gels formed at different scales suggest that the dimensions of the individual domains comprising the overall microstructure can be influenced simply by altering the size of the vessel in which they are formed. Changes in the localized mechanical properties as shown by cavitation rheology are seen as spherulite size changes. The pH-triggered low molecular weight gels investigated appear to consistently produce a uniform fibrous microstructure even when formed in vessels of different sizes, displaying consistent mechanical properties as a result.

From our results, we highlight the importance of carefully controlling parameters when preparing low molecular weight gels, particularly those formed via a solvent switch, as the microstructure and thus properties of these gels may be affected solely by the vessel and scale used to prepare samples for different characterization techniques and applications. We hope that this will encourage further insight into a previously unexplored aspect of producing these materials.

## Supporting Information Available

Full synthesis and characterization details, further SAXS data, CD data, TEM images and rheology data (PDF)

## Abbreviations

2NapFF: 2-naphthylmethyl ether diphenylalanine
2NapFV: 2-naphthylmethyl ether phenylalanine–valine
3D: three dimensional
DMSO: dimethyl sulfoxide
GdL: glucono-δ-lactone
H_2_O: water
LMWG: low molecular weight gelator
NaOH: sodium hydroxide
NMR: nuclear magnetic resonance
*P*_c_: critical pressure.
