# Supplementary material for: Effect of Imposing Spatial Constraints on Low Molecular Weight Gels
Source: Biomacromolecules. 2023 Aug 18;24(9):4253–62. doi: 10.1021/acs.biomac.3c00559 (PMC10498449; doi:10.1021/acs.biomac.3c00559)
Supplement: Supplementary file 1 — bm3c00559_si_001.pdf [file bm3c00559_si_001.pdf]

The effect of imposing spatial constraints on low molecular weight gels

Max J. S. Hill, Ana M. Fuentes-Caparrós and Dave J. Adams*
School of Chemistry, University of Glasgow, Glasgow, G12 8QQ, UK.

Supplementary Information

Spherulite Perimeters

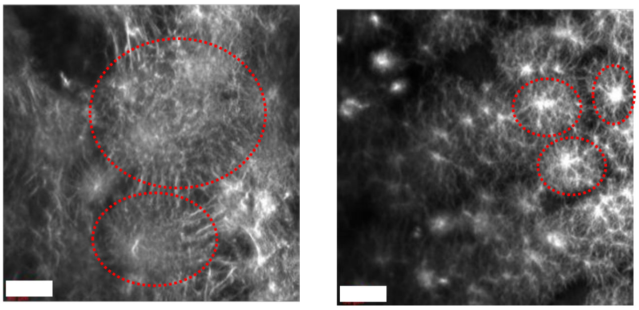

Figure S1. Example marked up confocal microscopy images of solvent triggered 2NapFV gels with examples of individual spherulites highlighted. Scale bars (white) = 50 μm.

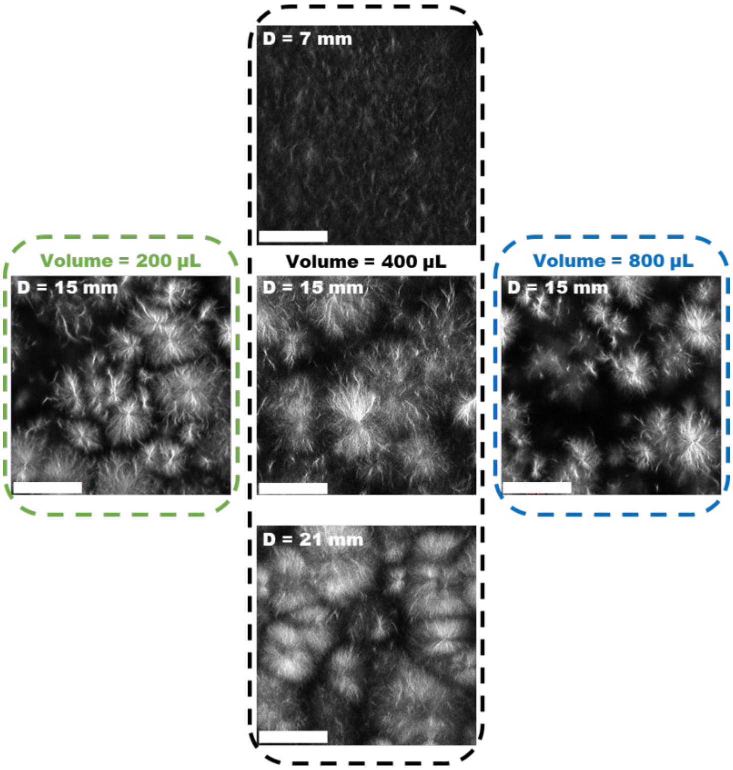

Figure S2. Confocal images of solvent triggered 2NapFF gels prepared in 3D printed ring-shaped vessels with 7mm (top), 15mm (middle) and 21 mm (bottom) diameters. All gels were prepared at 5 mg mL^{-1} using a φ_{DMSO} = 0.2. Sample volume was varied between 200 μL (left), 400 μL (centre) and 800 μL (right) for the gels formed in 15 mm diameter vessels to result in different height gels. Gels in 7 mm and 21 mm diameter vessels were formed at a volume of 400 μL. Nile Blue A dye was incorporated pre-gelation (0.1 wt% aqueous solution at 2 μL per mL of gel). Scale bars (white) represent 50 μm in all cases. D = vessel diameter.

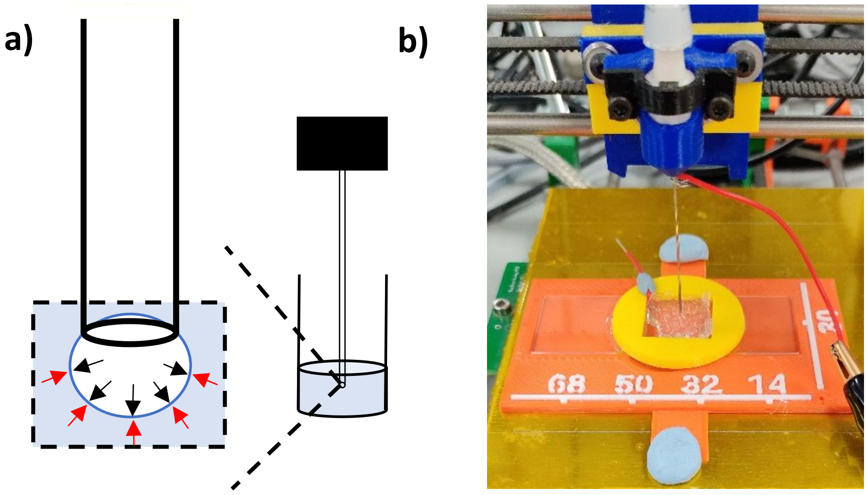

Figure S3. (a) Cartoon diagram of a cavitation bubble being formed within a gel. (b) Image of a 3D printed gel being analysed by cavitation rheology.

D (mm)	H (mm)	Perimeter (μm)	Deviation
7	10.39	173	13
8	7.96	129	19
9	6.29	284	133
10	5.09	799	167
11	4.21	1167	9
12	3.54	1045	-
13	3.01	828	-
14	2.60	780	148
15	2.26	610	47
16	1.99	766	-
17	1.76	493	36
18	1.57	354	48
19	1.41	604	-
20	1.27	815	-
21	1.15	598	149

Table S1. Perimeter of the spherulitic domains within solvent triggered 2NapFF gels of different thickness quantified using ImageJ.

D (mm)	H (mm)	Perimeter (μm)	Deviation
7	10.39	1153	114
8	7.96	713	79
9	6.29	465	19
10	5.09	466	53
11	4.21	707	37
12	3.54	482	39
13	3.01	860	77
14	2.60	739	52
15	2.26	666	54
16	1.99	1301	126
17	1.76	1207	157
18	1.57	1469	55
19	1.41	1243	139
20	1.27	1103	165
21	1.15	1263	123

Table S2. Perimeter of the spherulitic domains within solvent triggered 2NapFV gels of different thickness (quantified using ImageJ).
